# A comparative study of cystoid macula edema following glaucoma drainage device surgery versus trabeculectomy

**DOI:** 10.1007/s10792-024-03068-y

**Published:** 2024-03-20

**Authors:** Caroline Gietzelt, Lilo Koenig, Werner Adler, Friederike Schaub, Ludwig M. Heindl, Claus Cursiefen, Thomas S. Dietlein, Philip Enders

**Affiliations:** 1 Department of Ophthalmology, Faculty of Medicine and University Hospital Cologne, University of Cologne, Cologne, Germany; 2 Glaucoma Imaging Center, Faculty of Medicine and University Hospital Cologne, University of Cologne (GICC), Cologne, Germany; 3 Department of Medical Informatics, Biometry and Epidemiology, Friedrich-Alexander University Erlangen-Nuremberg, Erlangen, Germany; 4 Department of Ophthalmology, University Medical Centre Rostock, Rostock, Germany

**Keywords:** Glaucoma, Drainage device surgery, Trabeculectomy, Postoperative cystoid macular edema

## Abstract

**Purpose:**

To assess and compare the risk for development of cystoid macula edema (CME) after glaucoma drainage device (GDD) implantation versus conventional trabeculectomy with mitomycin (trab) for glaucoma.

**Methods:**

Retrospective review of consecutive patients receiving trab or GDD implantation between 2016 and 2018. Inclusion criteria were availability of pre- and postoperative spectral domain optical coherence tomography (SD-OCT) of the macula. SD-OCT images were evaluated for presence of CME qualitatively, central subfield thickness (CST) and macular volume (MV).

**Results:**

73 eyes could be included, 42 received trab and 31 GDD surgery. Eyes receiving trab on average had 0.8 ± 0.8 previous intraocular operations, while eyes with GDD implantation had 3.1 ± 1.9 (*p* < 0.001). Occurrence of postoperative CME was significantly more frequent after GDD implantation (6 out of 31 (19.4%)) than after trab (2 out of 42 eyes = 4.8%), (p = 0.049). Mean preoperative CST as well as MV was comparable in both groups (CST before trab: 282.7 ± 23.0 µm, CST before GDD 284.2 ± 27.3 µm, *p* = 0.287; MV before trab: 7.8 ± 1.1 mm^3^, MV before GDD: 8.0 ± 0.8mm^3^, *p* = 0.305). Mean postoperative CST and MV were significantly higher after GDD (CST 338.5 ± 129.3 µm, MV 8.8 ± 2.6 mm^3^) than after trabeculectomy (CST 290.6 ± 60.2 µm, *p* = 0.038; MV 7.8 ± 1.2mm^3^, *p* = 0.039).

**Conclusions:**

In real-life conditions, GDD surgery seems to be associated with a higher risk to develop CME when compared to conventional trabeculectomy. This information may be helpful for glaucoma surgeons to advise the patients on postoperative risks of surgery.

## Introduction

Trabeculectomy with mitomycin (MMC) and glaucoma drainage devices (GDD) both form a bypass from the anterior chamber to the subtennonal space where the aqueous humour is resorbed, in order to lower intraocular pressure (IOP) in glaucoma patients [1]. The use of GDDs has increased significantly over the past decades [2]. While the use of GDD’s as last resort of draining glaucoma surgery in eyes with high risk of failure or previously failed trabeculectomy is widely accepted, the use of primary GDD implantation instead of trabeculectomy with MMC has been discussed controversially. Different studies found no significant difference in the rate of surgical failure and occurrence of serious complications 3 years after surgery between the two procedures [3]. However, 5 years after surgery the success rate was significantly higher in the GDD group compared to the trabeculectomy group in the TVT study [4] leading to a discussion about GDD surgery as primary treatment option for glaucoma surgery.

Development of cystoid macula edema (CME) is a significant postoperative complication of all intraocular surgery leading to a reversible loss of visual acuity [5, 6]. After cataract surgery cystoid macular edema is called Irvine Gass Syndrome [7, 8] and is found in 4 to 20% of patients [9, 10]. After GDD surgery the frequency of CME was fount between 3.4 and 22% [11–13]. After trabeculectomy rates of occurrence of CME between 2.9% [13] and 8.7% [14] are reported.

From the existing literature, it is not completely clear whether CME is more frequent after GDD surgery than after trabeculectomy. The aim of this study was to assess real life data and compare the risk for development of postoperative CME in GDD surgery compared to trabeculectomy with MMC in primary open angle glaucoma, pseudoexfoliation and pigment dispersion glaucoma. Further aim was to describe qualitative and quantitative OCT features of CME.

## Materials and methods

Charts of all patients receiving conventional trabeculectomy with MMC in 2016 and of all patients receiving GDD implantation between 2016 and 2018 at the Department of Ophthalmology, Medical Faculty and University Hospital of Cologne, Germany were retrospectively reviewed. Data were retrieved from the patients' files: Clinical data on patients’ medical history including ophthalmologic diagnoses, previous eye surgery, best-corrected visual acuity (BCVA), topical medication, epidemiologic data, results of visual field testing and preoperative and postoperative high-resolution spectral domain optical coherence tomography (SD-OCT) imaging of the macula were collected from patients’ files. At our center we used Ahmed and Baerveldt implants as GDDs in the stated period [15]. Both implant types were routinely occluded by an absorbable suture to prevent hypotony in the early postoperative phase. Postoperative steroid and anti-inflammatory therapy protocols included use of dexamethasone and ofloxacin eye drops. In both surgical approaches, steroid eyedrops are reduced by approximately one drop per week depending on the remaining inflammation. In total, steroid eyedrops were given 4 to 6 weeks postoperatively. Specifications and details on surgical techniques and GDD implants used at the department have been described in previous publications of our group [16, 17].

### Inclusion and exclusion criteria

Inclusion criteria for this review were availability of pre- and postoperative SD-OCT of the macula. Patients with the following types of glaucomatous optic neuropathy were included: primary open angle glaucoma (POAG), pigment dispersion glaucoma (PDG) and pseudoexfoliation glaucoma (PXG) were included. Exclusion criteria were presence of other aetiologies of glaucoma, in particular cases with signs of inflammation or inflammatory ocular disease. Cases with insufficient image quality of the SD-OCT were excluded as well. Furthermore, patients with preoperative presence of CME or other pathologies affecting the accessibility of CME (e.g. nAMD, diabetic macular edema) were excluded.

### Retinal OCT imaging and diagnosis of CME

Retinal images of the macula were obtained by a SD-OCT (SPECTRALIS® HRA + OCT, Heidelberg Engineering, Heidelberg, Germany) platform and centered on the fovea. Two investigators (LK und PE) reviewed the images manually and checked qualitatively for presence or absence of CME. SD-OCT imaging in clinical routine used a pattern size of 20° × 15°, ART mode on with 21 images averages, a number of B-scans of 37 and a distance between B-Scans of 128 µm. Automated segmentation of retinal layers was controlled and correctly manually when necessary. Quantitative SD-OCT analysis comprised assessment of central subfield thickness (CST) and macular volume (MV). Therefore, the Early Treatment Diabetic Retinopathy Study (ETDRS) grid (6 mm × 6 mm square grid) was first manually replotted to the foveal centre, and errors in device-provided segmentation lines were corrected on all scans enclosed within the central subfield. CMT (μm) measurements were derived from the topographic map of the macular cube scan for a 1-mm foveal area, extracted from the ETDRS grid. MV (mm^3^) was also extracted from the topographic map of macular cube scan.

After a CME was detected with SD-OCT, the diagnosis of the etiology of CME as postoperative cystoid macular edema was made clinically. We made sure to exclude alternative reasons for a cystoid macular edema like macular neovascularisation (MNV) due to age related macular degeneration or other reasons, diabetic macular edema, epiretinal membranes or uveitis.

### Treatment of CME

Treatment options for CME after intraocular surgery in existing literature are topical non-steroidal anti-inflammatory agents (NSAIDs), topical steroids, peri-ocular or intra-ocular steroids or systemic steroids [18]. At our center, our clinical standard of care comprises hourly use of topical steroids for 1 week, follow by every two hours for one week. From the third week on, application of prednisolone eye drops is reduced by one drop weekly starting with 5 times a day.

### Statistics

Statistical analyses were conducted using the software SPSS (Version 25.0; IBM Corp, Armonk, New York, USA). The threshold for statistical significance was set to *p* < 0.05.

We tested for normal distribution using Shapiro–Wilk-Test. We used Student t test for paired and unpaired samples to compare means, and Chi-squared test to compare the occurrence of CME between the two surgical approaches GDD and trab. The relationship between the dependent variable CME and independent variables type of surgery (GDD vs. trabeculectomy) and number of previous surgeries was examined in a logistic regression model.

## Results

259 patients received conventional trabeculectomy with MMC in 2016 and 276 patients received GDD implantation between 2016 and 2018 at our center. Of those seventy-three eyes of seventy patients could be included, whereof 42 eyes received trabeculectomy with MMC and 31 eyes received surgery with GDD implantation. Reasons for exclusion were the absence of pre- or postoperative SD-OCT (n = 374, 70%), glaucoma diagnoses other than POAG, pigment dispersion glaucoma or PEX glaucoma (n = 54, 10%), preoperative presence of CME or other pathologies affecting the accessibility of CME (n = 57, 11%) and other reasons (n = 50, 27%).

Of the patients receiving GDD implants, 28 patients received a Baerveldt implant and three patients received an Ahmed implant in this study.

POAG was present in 54 eyes (74.0%), pseudoexfoliation glaucoma in 18 eyes (24.7%) and pigment dispersion glaucoma in one eye (1.4%). Intraocular pressure at baseline was 21.6 ± 8.1 mmHg before trabeculectomy with MMC and 24.0 ± 7.1 mmHg before GDD implantation (p = 0.200). No patient with postoperative CME had a relevant epiretinal gliosis. One patient, who developed CME after GDD, had diabetes without diabetic macular edema. All other patients, who developed CME after GDD and all patients who developed CME after trabeculectomy had no diabetes. Preoperative SD-OCT took place in average 43.8 ± 73.9 days before surgery. All available postoperative SD-OCT images of each patient were reviewed up to 2 years after surgery. The SD-OCT image used for quantitative analysis was obtained in average 167.2 ± 105.5 days after surgery. The time span between surgery and OCT examination was not significantly different for the two groups trabeculectomy and GDD (time after surgery trabeculectomy = 169.0 ± 90.1 days; time after GDD implantation = 164.8 ± 124.9 days; *p* = 0.869). Table 1 summarizes epidemiological data.

**Table 1:** Epidemiologic data

	Trabeculectomy	GDD
**Age (years)**		
Mean ± SD	66.6 ± 11.7	66.1 ± 14.1
Range	28 to 85	25 to 58
**Glaucoma diagnosis**		
Primary open-angle glaucoma	29 (69)	25 (81)
Pseudoexfoliation glaucoma	13 (31)	5 (16)
Pigment dispersioin glaucoma	0	1 (3)
**Spherical equivalent at baseline (dpt)**		
Mean ± SD	− 1.3 ± 3.2	− 0.8 ± 1.3
Range	− 11.0 to + 3.4	− 4.75 to 1.5
**IOP at baseline (mm Hg)**		
Mean ± SD	21.6 ± 8.1	24.0 ± 7.1
Range	9 to 40	8 to 40
**Number of previous intraocular surgeries**		
Mean ± SD	0.8 ± 0.8	3.1 ± 1.9
Range	0 to 4	1 to 8
**Mean deviation in TOP perimetry at baseline**		
Mean ± SD	− 9.9 ± 9.7	− 12.0 ± 5.9
Range	− 26.2 to 13.7	− 22.6 to -0.3

Mean IOP in eyes with postoperative CME was not significantly different between trabeculectomy and GDD group (IOP at time point of detection of postoperative CME (trab) = 13.5 ± 4.9 mmHg; IOP (GDD) = 12.2 ± 7.6 mmHg; *p* = 0.289).

Postoperative comlications occurred in 15 of all 73 patients. Table 2 details postoperative complications in the different groups.

**Table 2:** Postoperative complications in both groups

	Postoperative hypotony, injection of healon into anterior chamber or insertion of additional suture	Postoperative elevated IOP, revision surgery necessary	Conjunctival dehiscence, surgery necessary	Shortening of tube necessary	Vitrectomy because of malignant block	no postoperative complications
GDD without CME	2	2	1	–	–	20
GDD with CME	–	–	–	1	–	5
TE without CME	4	4	–	–	–	32
TE with CME	–	–	–	–	1	1

### Postoperative central subfield thickness (CST) and macular volume (MV) and occurrence of CME

**Fig. 1:**
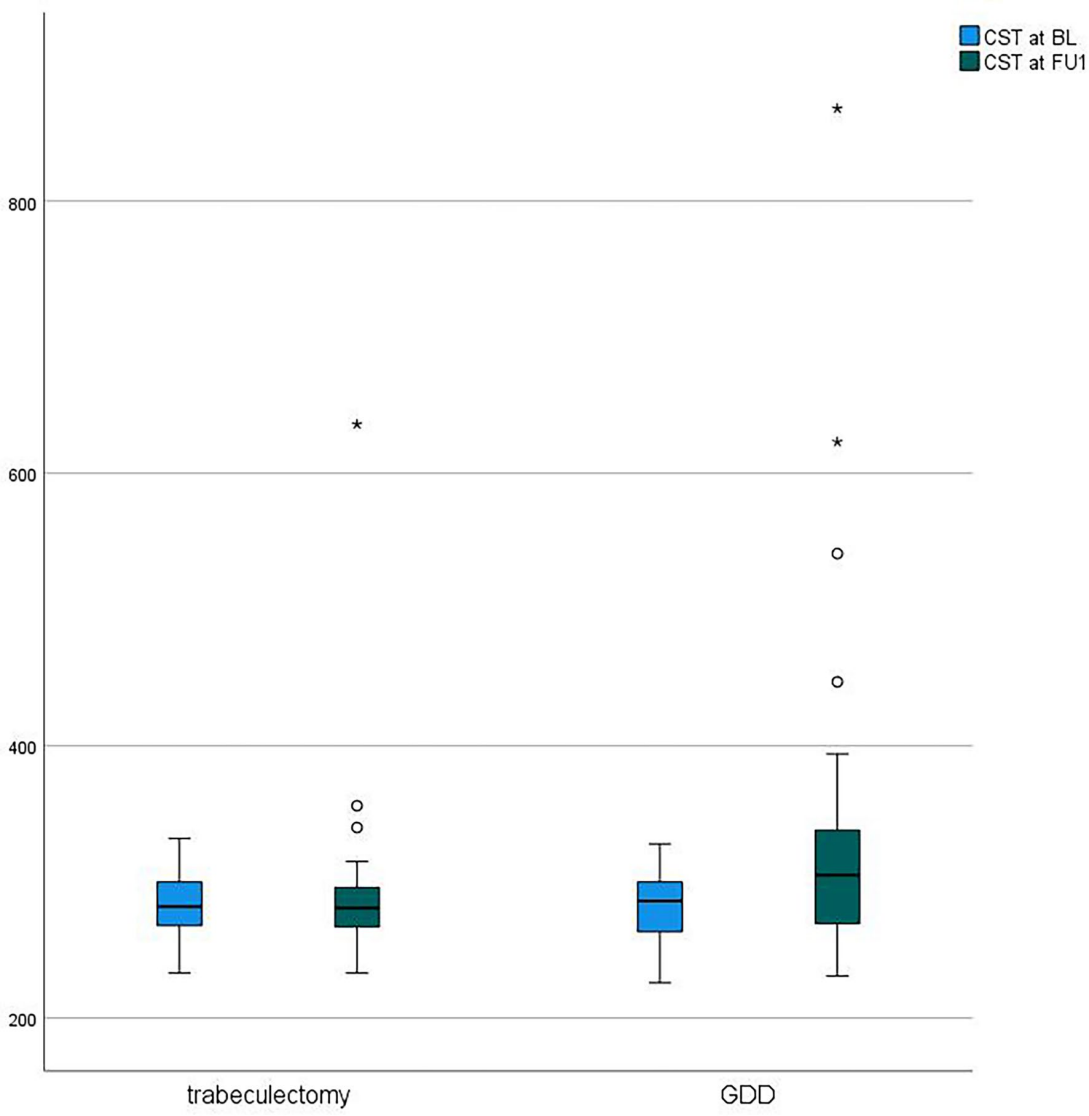
Boxplot diagram showing central subfield thickness (CST) at baseline (BL) and first postoperative follow up (FU1) comparing trabeculectomy with mitomycin c and GDD surgery. Preoperative CST was comparable between both groups. CST increased significantly from BL to the first follow up (FU1) in the GDD group. There was no significant increase in CST in the trabeculectomy group from BL to FU1

Mean preoperative CST in patients receiving trabeculectomy with MCC was 282.7 ± 23.0 µm and thereby comparable to CST in the GDD group with 284.2 ± 27.3 µm (*p* = 0.796). In the GDD group, CST increased significantly from 284.2 ± 27.3 µm at baseline to 338.5 ± 129.3 µm at the postoperative follow-up (*p* = 0.028). In the trabeculectomy group, there was no significant increase in CST between baseline and the follow-up exam (postoperative CST = 290.6 ± 60.2 µm; *p* = 0.305). The CST was significantly higher after GDD than after trabeculectomy in our study (*p* = 0.038). Figure 1 shows boxplot diagrams of CST at baseline and follow-up comparing trabeculectomy and GDD surgery.

**Fig. 2:**
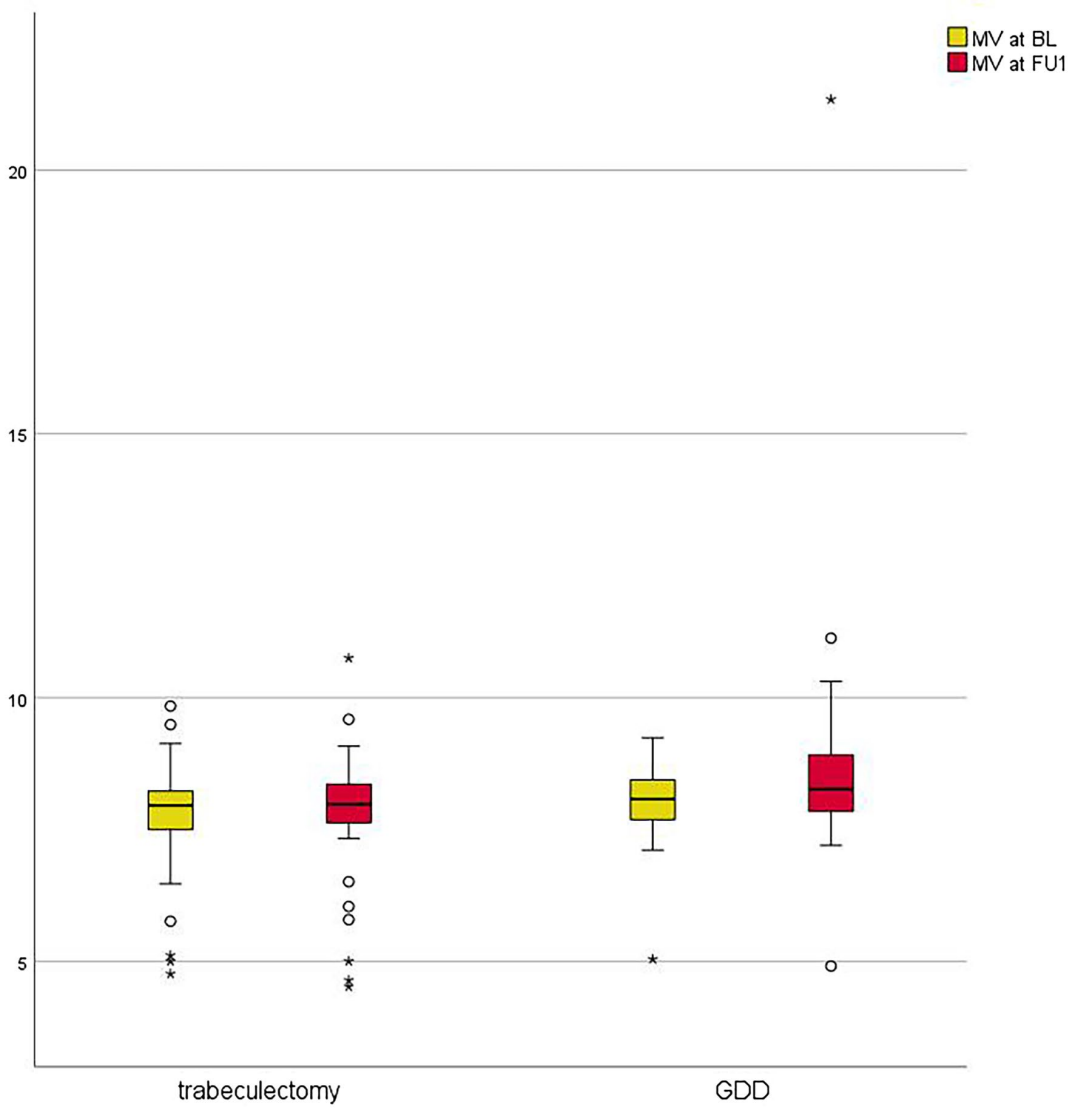
Boxplot diagram showing macular volume (MV) at baseline (BL) and first postoperative follow up (FU1) comparing trabeculectomy with mitomycin c and GDD surgery. Preoperative MV was comparable between the two groups. Mean MV did not change significantly after trabeculectomy, but after GDD mean MV increased significantly

Mean preoperative MV was 7.8 ± 1.1 mm^3^ in the trabeculectomy group and 8.0 ± 0.8mm^3^ in the GDD group. These preoperative values were comparable between the two groups (*p* = 0.264). Mean MV did not change significantly after trabeculectomy (postoperative MV = 7.8 ± 1.2 mm^3^; *p* = 0.777). After GDD there was an increase to 8.8 ± 2.6 mm^3^, which however also did not reach the level of statistical significance (*p* = 0.121). Postoperative MV in the GDD group was significantly higher compared to the trabeculectomy group (*p* = 0.039). Figure 2 shows boxplot diagrams of MV at baseline and follow-up comparing trabeculectomy and GDD surgery.

After trabeculectomy with MMC (trab), 4.8% of eyes developed CME while 19.4% of eyes after GDD implantation showed postoperative CME. This fourfold higher share of CME in GDD was statistically significant (*p* = 0.049).

Of the six patients who developed CME after GDD, one patient had received an Ahmed implant, the other five had received a Baerveldt implant.

Eyes receiving trabeculectomy with MMC on average had 0.8 ± 0.8 previous intraocular operations, while eyes with GDD implantation had 3.1 ± 1.9 (Mann–Whitney-U Test *p* < 0.001). Eyes with occurrence of postoperative CME in both groups (n = 8) had on average 2.5 ± 1.8 intraocular operations, those without CME (n = 61) had 1.7 ± 1.7 (Mann–Whitney-U Test *p* = 0.12).

A logistic regression model examined the relationship between the dependent variable CME and independent variables type of surgery (GDD vs. trabeculectomy) and number of previous surgeries. Table 3 displays the results. Both independent variables are non-significant (type of surgery *p* = 0.117; number of previous surgeries *p* = 0.962).

**Table 3:** Relationship between the dependent variable CME and independent variables type of surgery (GDD vs. trabeculectomy) and number of previous surgeries in a logistic regression model

	OR	95% CI	p
Type of surgery (GDD)	4.929	0.708; 44.629	0.117
number of previous surgeries	0.988	0.579; 1.555	0.962

Of the six patients, who developed CME after GDD, five still had IOP-lowering eyedrops (four of them with prostaglandin analogs amongst the medication, one of them without) at the time of detection of the CME. Only one patient with CME after GDD had no IOP-lowering medications at the time of detection of the CME.

### Clinical course and treatment of CME

In eyes with CME in our study, best-corrected visual acuity decreased in average by four lines after development of CME (mean BCVA before surgery = 0.32 logMAR, mean BCVA with CME = 0.84 logMAR). CME in all cases was treated topically according to the clinical standard at our center as described in the methods section.

Follow-up data for ≥ 24 months after surgery was available only in six eyes with postoperative CME in our cohort. The other eyes had a loss to follow-up before.

In three of the six cases with follow-up, CME resolved completely or nearly completely under medication with topical prednisolone, in one case already after four weeks, in two cases after 2 and 3 years respectively after surgery.

In the other three cases however, CME persisted despite treatment until the most recent follow up examination 2 to 5 years after surgery. Of those three cases, the decreased visual acuity recovered completely despite persistence of the CME in only one case, in one case the decreased visual acuity recovered partially and in one case the decrease in visual acuity persists up to now 5 years after surgery.

**Fig. 3:**
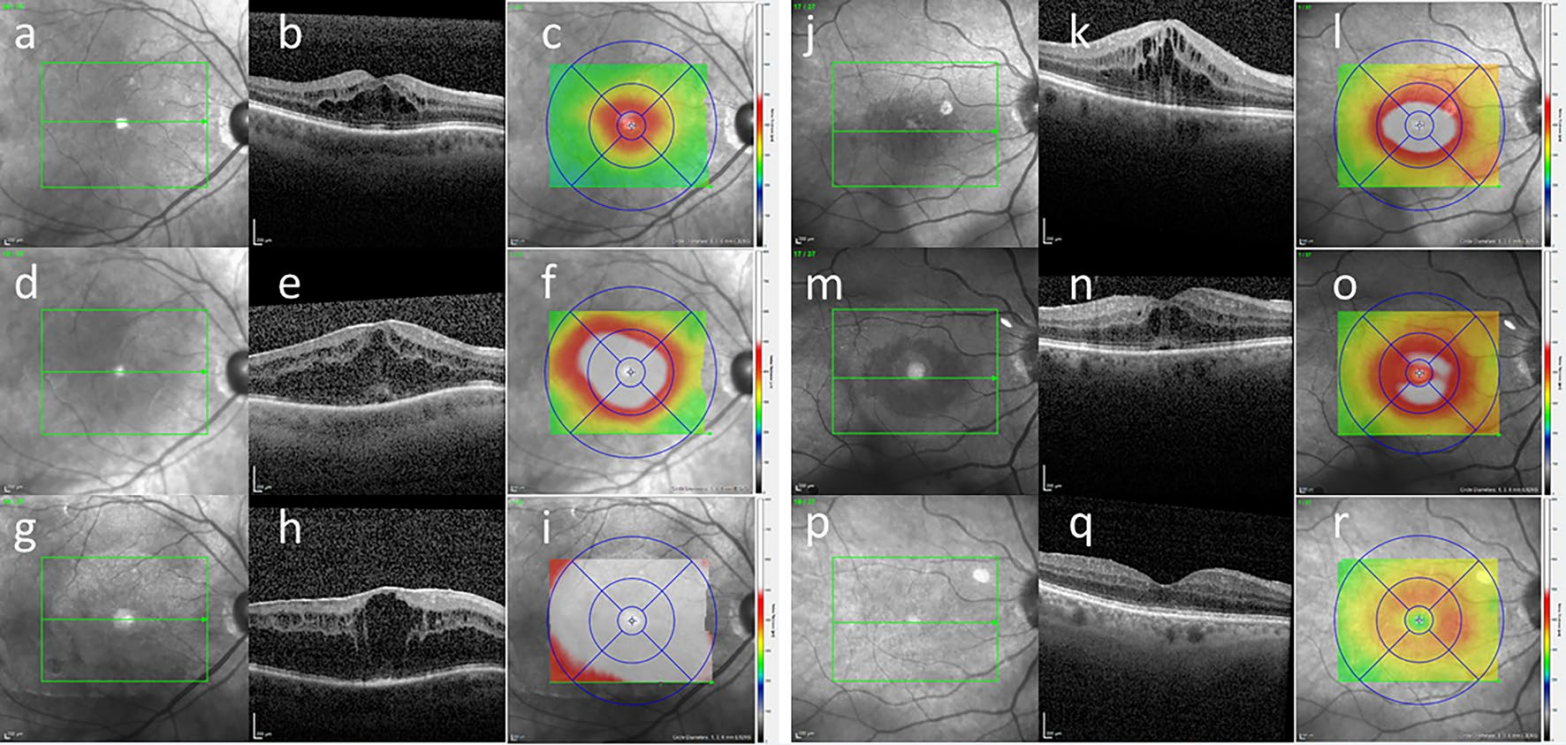
SD-OCT examinations in two eyes with CME after GDD or trabeculectomy. Patient with CME after GDD surgery 62 days after surgery (**a**–**c**), 216 days after surgery (**d**–**f**), and persisting CME 2 years after surgery (**g**–**i**) and patient with CME after trabeculectomy surgery 15 days after surgery (**j**–**l**), persisting CME 2 years after surgery (**m**–**o**), and resolved CME 3 years after surgery (**p**–**r**). The en face views (**a**, **d**, **g**, **j**, **m**, **o**) give an overview and show the incision of sectional images (**b**, **e**, **h**, **k**, **n**, **q**). Topographic mappings of macular thickness with ETDRS grids are given (**c**, **i**, **h**, **l**, **o**, **r**)

Figure 3 shows SD-OCT pictures of two patients with CME after GDD surgery and trabeculectomy over time.

## Discussion

In our study, postoperative CST and MV were significantly higher after GDD than after trabeculectomy in our study (*p* ≤ 0.039 respectively). Also, CME occurred nearly four times more frequently after GDD implantation compared to trabeculectomy, this difference was statistically significant.

Postoperative CME occured in 4.8% of eyes undergoing trabeculectomy with MMC. This frequency seems consistent with existing evidence for CME after trabeculectomy and comparable to the incidence of CME after cataract surgery. Previous studies described the incidence of postoperative CME to range between 3 and 9% after trabeculectomy with MMC [11–14] and between 4 and 20% for cataract surgery [9, 10]. Differences in study design, length of follow-up or inclusion criteria might explain the relevant range of CME incidence rates in these studies. Some comprised of a twelve months follow-up while others included a follow-up of up to 5 years. The risk of CME occurrence after trabeculectomy has been linked to the type of glaucoma. Manabe et al. found pseudoexfoliation glaucoma to increase the risk of CME compared to POAG [14]. Also, the number of previous surgical procedures and comorbidities varied between different study cohorts.

The risk for postoperative CME seems to be relevantly higher than after non-penetrating glaucoma surgery like 360° suture trabeculotomy or canaloplasty. In those surgical procedures a recent study found postoperative CME to occur after 0.7 and 0.3% of stand alone surgeries respectively [19].

Eyes undergoing GDD surgery had a CME rate of 19.4% in our study. This matches existing evidence, where rates of CME formation between 3 and 22% after GDD implantation have been reported [11–13]. In a retrospective study on 185 glaucomatous eyes, Bhakta and colleagues described a rate of 22% for occurrence of visually significant CME after GDD implantation. This rate was significantly associated with the number of previous intraocular surgeries [13]. This study included eyes with inflammatory ocular disease (i.e. iritis) and was restricted to eyes with the occurrence of visually significant CME only.

The mechanisms of postoperative formation of CME have not yet been fully understood. A sterile inflammation resulting an insufficiency of the blood–aqueous barrier is discussed [6]. Surgical trauma seems to lead to a release of inflammatory mediators as well as free radicals [20]. This leads to a breakdown of the blood-aqueous barrier, which is indicated by an elevated level of laser flare in the anterior chamber in eyes with postoperative CME [21]. As a result, the vascular permeability is increased, and fluid accumulates in extracellular spaces of the perifoveal retina [20]. Many other factors have been described to be associated with a higher risk of postoperative CME, these include a history of uveitis [22], the history of previous pars plana vitrectomy for retinal detachment as well as the preexistence of epiretinal membranes (ERM) [23]. Also topical prostaglandin analogs may promote macular edema [24, 25]. While the risk of postoperative formation of CME seems not increased due to glaucoma alone [26] and also ab interno glaucoma surgery combined with cataract surgery does not seem to have an elevated risk compared to stand alone cataract surgery [27], there is evidence for an increased risk of postoperative formation of CME after GDD surgery to treat glaucoma [12]. Also the type of glaucoma seems to have an influence on the rate of formation of CME as Manabe et al. reported significantly higher rates of CME after trabeculectomy in pseudoexfoliation glaucoma than in POAG [14]. The frequency of CME formation shows some discrepancy between real life data and data from clinical trials. [12, 13]

The larger surgical trauma and an increased inflammatory response to the medical implant could be two reasons explaining the increased risk for CME development in GDD surgery as well as significantly higher CST after GDD.

Prior to surgery, 91% of eyes were treated with prostaglandin analogs in our study (95% before trabeculectomy, 87% before GDD). Potentially this could also have increased the risk of postoperative CME compared to surgery in non-glaucomatous eyes. We found no significant difference in the occurrence of CME between eyes, which were previously treated with prostaglandin analogs and eyes, which were not. This is in line with a recent study by Fakhraie et al. who found no increased risk for postoperative CME after cataract surgery in patients treated with latanoprost [28, 29].

Postoperative CME can lead to a significant reversible or non-reversible loss in visual acuity [30].

Interestingly, recently our group reported that intense, early postoperative topical steroid therapy could significantly reduce the CME risk after posterior lamellar grafting combined with phacoemulsification [31]. In eyes receiving prednisolone eye drops hourly for the first postoperative week the CME rate was 0% versus 12% in eye just receiving 5x/day (p < 0.05). That potentially opens a new avenue for prevention of CME also in eyes after glaucoma surgery [31].

Limitations of our study include the retrospective set up of the study. The number of study eyes and strict inclusion and exclusion criteria reduce this effect.

Due to the strict exclusion criteria only 73 patients out of 535 screened patients could be included in the study. This was mainly due to other glaucoma diagnoses, but also due to missing preop or postop SD-OCT. This could depict a potential bias, as those patients with full visual acuity not always receive a SD-OCT. Also, patients with postoperative complications are referred more often to the tertiary center for follow-up. These two factors could have led to an overestimation of CME in our cohort. This applies to both groups and therefore should not bias the direct comparison on the frequency of CME occurrence.

Due to the use of real life data, our study falls short to allow a clear differentiation between the variables number of previous surgeries and type of surgical procedure regarding their impact on CME occurrence and on quantitative OCT measurements.

A logistic regression model did not reach the threshold for statistical significance; however, a tendency towards a higher relevance of type of surgery may be deducted from the variable’s higher odds ratio and 95% confidence interval. Furthermore, we did not find a significant correlation between the number of previous intraocular surgeries and the occurrence of a CME (*p* = 0.235). The conditions of this study represent conditions in real life, where patients receiving GDD often have experienced a greater number of previous surgeries. To differentiate further between the influence of the number of previous surgeries and the type of surgical procedure, a prospective study would be necessary including only patients who receive trab or GDD as primary surgical intervention for glaucoma.

In conclusion, CME is a relevant complication after both trabeculectomy and GDD surgery. GDD surgery as primary surgical option for non-uveitic open angle glaucoma is discussed controversially. At our center we use GDD only as treatment option for eyes with high risk of failure or previously failed trabeculectomy with MMC.

This study found, that in real life CME occurred significantly more often after GDD in patients with a higher number of previous surgeries than after trab in patients with a lower number of previous surgeries. This may represent a relevant information for the choice of surgical treatment and preoperative risk assessment.
